# Effect of Depressive Symptoms on Return to Work After Lumbar Spine Fusion Surgery: A Retrospective Registry Study

**DOI:** 10.1097/BRS.0000000000005469

**Published:** 2025-10-24

**Authors:** Jenna L.C. Laurén, Leevi A. Toivonen, Jussi P. Repo, Hannu Kautiainen, Marko H. Neva

**Affiliations:** a Department of Surgery, Hospital Nova of Central Finland, Jyväskylä, Finland; b Department of Orthopaedicsand Trauma, Tampere University Hospital, Tampere, Finland; c Faculty of Medicine andHealth Technology, Tampere University, Tampere, Finland; d Primary Health Care Unit,Kuopio University Hospital, Kuopio, Finland; e Folkhälsan Research Center, Helsinki, Finland

**Keywords:** depressive symptoms, lumbar spine fusion, major depressive disorder, MDD, MHI-5, return to work, RTW

## Abstract

**Study Design.:**

Retrospective registry study.

**Objective.:**

To investigate the influence of depressive symptoms on return to work (RTW) after lumbar spine fusion (LSF) surgery over a two-year follow-up. A secondary objective was to evaluate the development of depressive symptoms separately in patients who succeeded or failed to RTW after surgery.

**Background.:**

Depressive symptoms are overrepresented in patients undergoing LSF surgery. The effect of depressive symptoms on RTW after LSF surgery has not yet been widely studied.

**Materials and Methods.:**

A total of 348 consecutive patients available to the workforce underwent LSF surgery. Depressive symptoms were evaluated at baseline and three, 12, and 24 months postoperatively using the MHI-5. Lower scores have been shown to indicate greater depressive symptoms, and a cutoff score of ≤68 indicates current major depressive disorder (MDD). Cumulative RTW and MHI-5 scores were prospectively followed up at the aforementioned time points.

**Results.:**

Before surgery, almost half (46%) of the patients had MDD. The presence of preoperative MDD indicated a lower RTW at two years [72% *vs*. 82%, adjusted HR: 0.77 (95% CI: 0.61 to 0.98)]. The probability of RTW was correlated with an increase in the MHI-5 score until the inflection point of 70, after which a further increase in the MHI-5 score no longer increased the probability of RTW. In addition, the MHI-5 score was significantly higher than baseline at three months postoperatively [13 points (95% CI: 10 to 15)] and remained higher in patients who RTW during two years. However, only a marginal increase in MHI-5 was observed in those who failed to RTW. This finding indicates fewer depressive symptoms in patients who successfully RTW.

**Conclusion.:**

Patients with preoperative MDD were less likely to RTW after LSF surgery. After surgery, depressive symptoms diminished in the group who did successfully RTW.

**Level of Evidence.:**

Level III.

The world’s leading nonfatal causes of disability are depression and back pain.^1^ In fact, the combination of chronic back pain and major depression is linked to greater disability than either condition on its own.^2^ Patients with chronic pain, particularly chronic back pain, are more likely to experience depressive symptoms.^2–6^ The risk of major depression is related to back pain severity.^2^ The Mental Health Inventory (MHI-5), a subset of the Short Form Health Survey (SF-36), is easily available and can be used to identify major depressive disorder in adults.^7^ Such an accessible measure is practical for longitudinal evaluations in research settings, unlike the gold standard, diagnosis made by a psychiatrist.

Return to work (RTW) is both a primary goal of surgery and an important measure of its success. Postoperative RTW rates after lumbar spine fusion (LSF) surgery range from 39% to 92% depending on patient selection, patients’ comorbidities, preoperative work status and follow-up time.^8–11^ Inability to work leads to productivity loss, imposing a high economic burden on both the individual patient^12–14^ and the employers.^14,15^ The incidence of spinal fusions has increased over the last few decades.^16–18^ One main target of spinal fusion is to preserve the working capacity of employees. The prevalence of depressive symptoms in patients undergoing lumbar fusion for degenerative spine disease has been reported at 15% to 35% preoperatively.^19–21^ Although the prevalence of depressive symptoms has been shown to decrease after surgery to 11% to 24%,^19–21^ it is still higher than that in the average European population with a prevalence of 6.4%.^22^

Poorer patient-reported outcomes, such as higher postoperative pain and functional disability, have been reported after lumbar spine surgery^23^ and lumbar fusion surgery^20,24^ in patients with increased depressive symptoms. Patients with preoperative depressive symptoms have also been found to RTW at lower rates after lumbar fusion surgery.^11,25^

The aim of this study was to investigate the influence of major depressive disorder on RTW after LSF surgery in a two-year follow-up. A secondary objective was to evaluate the development of depressive symptoms separately in patients who succeeded or failed to RTW after surgery.

## MATERIALS AND METHODS

### Patients

All patients undergoing elective LSF during years 2008 to 2014 in Tampere University Hospital and Central Finland Central Hospital were invited to participate in a prospectively collected study. At the time of data collection, information on 817 patients was available. During the study period, these two tertiary hospitals were the only units performing LSF in their geographical catchment area of 775,000 inhabitants. Orthopedic surgeons recruited the patients at the outpatient clinic, after the decision to perform surgery. At the time of surgery, the surgeon filled in data on the indications for surgery, predominant symptoms, and surgical details. All surgeries included pedicle screw instrumentation and decompression through posterior, midline approach. Interbody devices were used at the surgeon’s discretion. According to normal clinical practice, follow-up was arranged with postoperative visits to the surgeon at three months and one year after surgery. This study included patients who were available to the workforce (18–65 yr). Patients not currently working, such as full-time students and homemakers, have been included into study, as they were thought to be able to take part in work-life. Patients on disability pension or on retirement at the time of surgery were excluded from the study. Cumulative return to work was retrospectively collected from patient records with no missing data. Data on MHI-5 scores were available at baseline and at 24 months for 348 (100%) and 296 (85%) patients, respectively. During the study period, a common practice was to issue a medical certificate allowing sick leave for three months postoperatively, thereafter, sick leave was continued if needed. The exclusion criteria were surgery for a tumor or acute fracture. Socioeconomic statuses were derived from patient-reported job titles in the baseline questionnaires, based on the Classification of Occupations 1989 in Finland.^26^

### MHI-5 and Major Depressive Disorder

The Mental Health Inventory Survey (MHI-5) is a brief, 5-item subset of the Short Form Health Survey (SF-36).^27^ The key features of the MHI-5 are five questions that measure various aspects of mental health in the past four weeks, including anxiety, depression, and psychological distress. Response options range from “all of the time” to “at no time,” and are scored on a six-point scale (altogether scores from 0 to 100), with higher scores indicating better mental health. The MHI-5 has been validated as an effective screening tool for identifying individuals with major depressive disorder (MDD) in the general adult population in the past four weeks.^7^ The optimal threshold in MHI-5 for identifying MDD as defined in Diagnostic and Statistical Manual of Mental Disorders (DSM-5)^28^ has been suggested to be ≤68, with associated sensitivity of 0.85 and specificity of 0.88.^7^ In the present study, patients were categorized according to this threshold into MDD- (MHI-5 >68, patients without major depressive disorder) and MDD+ (MHI-5 ≤68, patiens with major depressive disorder) groups. The MHI-5 scores were derived from the patients’ responses to SF-36 before the surgery and at three, 12, and 24 months postoperatively.

### Pain and Disability

A questionnaire including patient-reported outcome measures (PROMs) was administered before surgery and at three, 12, and 24 months postoperatively. The Visual Analog Scale (VAS) for back and leg pain was included, with scores ranging from 0 to 100 mm, with higher scores indicating greater pain.^29^ The Oswestry Disability Index (ODI),^30,31^ which ranges from 0 to 100, with higher scores reflecting greater disability, was also incorporated.

### Statistical Analysis

Patient characteristics and clinical data are expressed as mean and SD, median and interquartile range (IQR), or count and percentage (%), as appropriate. Time-to-event analysis for RTW was based on the product limit estimate (Kaplan-Meier) of the cumulative function. Differences between MDD groups were evaluated using unpaired Student *t* test, permutation test, Mann-Whitney *U* test, two-way analysis of variance, χ^2^ test, or Fisher exact test, as appropriate. Hazard ratios (HRs) for RTW were calculated using univariate and multivariate Cox proportional hazard models. A possible nonlinear relationship between the MHI-5 score and RTW was assessed by using the 3-knot-restricted cubic spline Cox proportional hazards model. A *P*-value of 0.05 was considered statistically significant. Statistical package Stata 18.0 StataCorp LP (College Station, TX, USA) was used for the analyses.

### Ethics, Funding, and Potential Conflicts of Interest

All participants provided a written informed consent to participate in the study. The study was approved by the Ethical Boards of Tampere University Hospital and Central Finland Central Hospital. The authors declare no conflicts of interest. The State funding for university-level health research, Tampere University Hospital, Wellbeing Services of Pirkanmaa has supported this study financially. The author (J.L.C.L.) also utilized independent research funds for thesis work from the Vappu Uuspää Foundation and The Paulo Foundation, Finland. The STROBE reporting guideline was followed in this study.^32^

## RESULTS

Of all patients in the database (n = 817), 348 were available to the workforce (age 18–65 yr) and met the inclusion criteria and were included in the study (Table 1). Of these 348 patients, 190 (55%) were working preoperatively. The mean age at the time of surgery was 50 years. Of these patients, 58% were women. The median duration of the spinal complaints was 10 years. Almost half of the patients (44%) were low-level employees, and one third (34%) were considered manual workers (“blue collar”). The most common indications for surgery were degenerative spondylolisthesis (34%) and isthmic spondylolisthesis (30%). All patients underwent posterolateral instrumented fusion, where most of the fusions (87%) were short (one–two levels).

**TABLE 1:** Patient Characteristics and Clinical Data

	MDD+(MHI-5 ≤68); N = 160	MDD-(MHI-5 >68); N = 188	*P*
Women, n (%)	93 (58)	108 (57)	0.90
Age, yr, mean (SD)	50 (9)	50 (9)	0.90
Education years, mean (SD)	13.4 (3.5)	13.2 (3.2)	0.63
Smoking, n (%)	38 (24)	38 (20)	0.43
Units of alcohol per week, median (IQR)	2 (0.6)	2 (0.6)	0.39
Cohabiting*, n (%)	74 (72)	98 (78)	0.98
BMI, mean (SD)	27.1 (4.1)	27.4 (4.0)	0.45
Socioeconomic group, n (%)			0.52
Self-employed persons	9 (6)	15 (8)	—
Upper-level employees	23 (14)	29 (15)	—
Lower-level employees	74 (46)	79 (42)	—
Manual workers, “blue collar”	54 (34)	65 (35)	—
Self-rated physical demand of work, n (%)			0.19
Light/nondemanding	75 (47)	74 (39)	—
Moderately demanding	41 (26)	54 (29)	—
Demanding	44 (28)	60 (32)	—
Sick leave, d, median (IQR)†	60 (5 to 180)	109 (7 to 285)	0.024
Working at the time of surgery, n (%)	99 (62)	91 (48)	0.012
Duration of the spinal problem, yr, median (IQR)	7.8 (3.0, 15.0)	10.0 (3.0, 20.0)	0.36
VAS-BP, mean (SD)	60 (25)	66 (22)	0.017
VAS-LP, mean (SD)	60 (26)	64 (24)	0.096
ODI, mean (SD)	38 (14)	47 (14)	<0.001
Comorbidities, n (%)			
Cardiovascular	61 (38)	64 (34)	0.43
Diabetes	9 (6)	15 (8)	0.39
Rheumatoid	3 (2)	4 (2)	0.98
Neurological	5 (3)	2 (1)	0.25
Psychiatric	2 (1)	8 (4)	0.12
Respiratory	3 (2)	7 (4)	0.35
Indication for surgery, n (%)			0.12
Degenerative spondylolisthesis	56 (35)	63 (34)	—
Isthmic spondylolisthesis	54 (34)	49 (26)	—
Degenerative disc disease	21 (13)	30 (16)	—
Spinal stenosis	12 (8)	24 (13)	—
Deformity	11 (7)	7 (4)	—
Postoperative conditions	6 (4)	15 (8)	—
Previously undergone spinal surgery, n (%)	37 (25)	58 (32)	0.142
Fusion length, levels, n (%)			0.72
1–2	140 (88)	162 (86)	—
>2	20 (13)	26 (14)	—

Patients are divided into groups according to preoperative MHI-5 scores, with threshold 68 points indicating current major depressive disorder (MDD).

* Domestic relationship or marriage.

† Sick leave days during the last 12 months before surgery.

BMI indicates body mass index; BP, back pain; LP, leg pain; ODI, Oswestry Disability Index; VAS, Visual Analog Scale.

The mean preoperative MHI-5 score was 65. Based on the MDD threshold of 68, 160 (46%) patients were included in the MDD+ group and 188 (54%) in the MDD- group (Table 1). Both groups were similar in size, age, sex, and self-reported comorbidities. Patients in the MDD+ group had more severe preoperative disability (ODI 38 *vs*. 47 points) and back pain (VAS-BP 66 *vs*. 60 points; Table 1).

### Return to Work and Major Depressive Disorder

**Figure 1:**
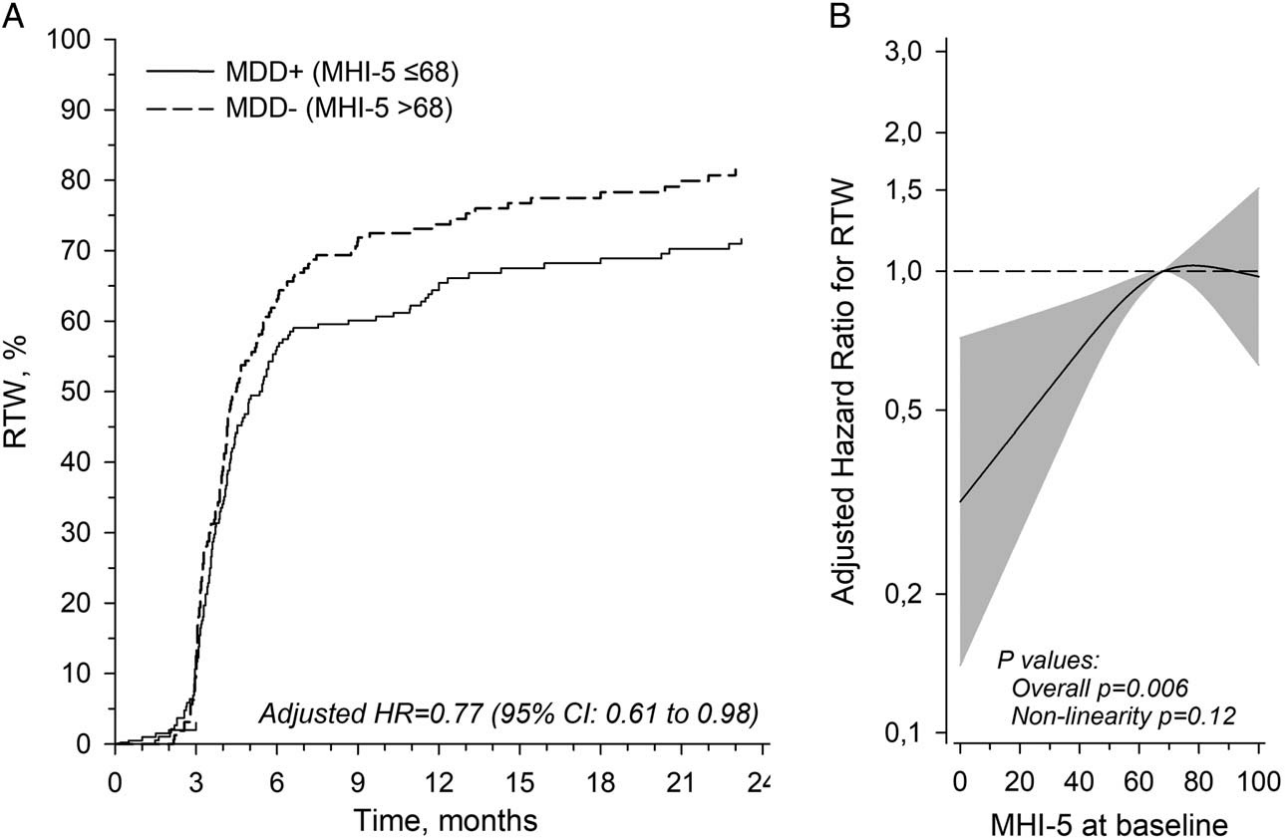
A, Kaplan-Meier estimates for RTW. Adjusted relationships at different scores of MHI-5. Age, sex and education years adjusted. B, Relationships of RTW as the function of the MHI-5. The curves were derived from a 3-knot restricted cubic splines Cox regression models. The model was age, sex, and education years. The gray area represents 95% CIs. Denominator of sHR was 68 level of MHI-5.

The overall RTW rate at 12 months was 69% (95% CI: 64 to 73) and 76% (95% CI: 71 to 81) at 24 months (Figure 1A). The MDD+ group was less likely to RTW [adjusted hazard ratio: 0.77 (95% CI: 0.61 to 0.98)]. In the MDD+ group, RTW at 12 and 24 months was 65% (95% CI: 59 to 72) and 72% (95% CI: 65 to 78), respectively. In the group MDD-, RTW at 12 and 24 months was 74% (95% CI: 67 to 80) and 82% (95% CI: 75 to 87), respectively. When analyzing RTW per continuous MHI-5 scores, the likelihood of RTW increased linearly until the threshold of 68 in MHI-5, after which the likelihood of RTW remained stable (Figure 1B).

**Figure 2:**
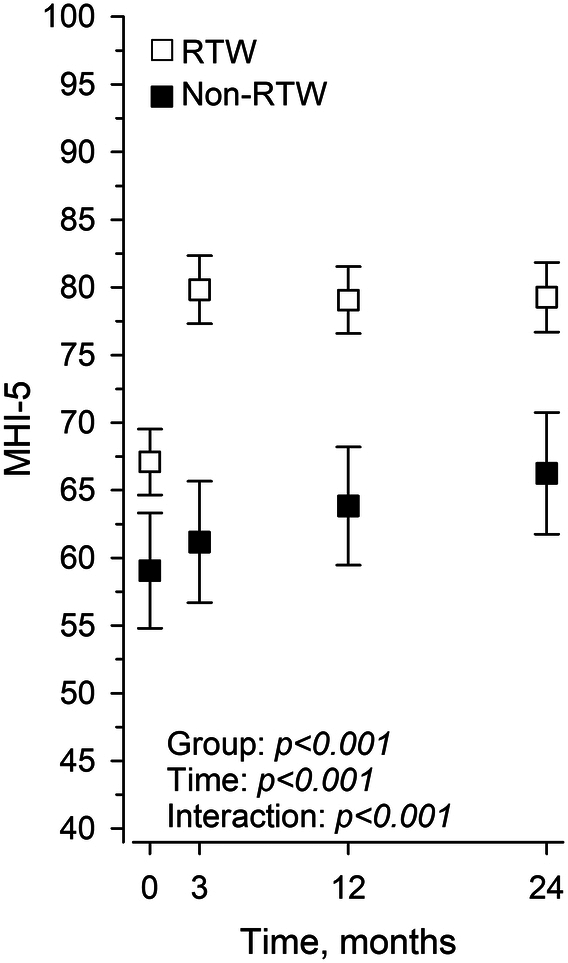
MHI-5 scores in patients who RTW and those who do not. Groups adjusted by age, sex and education years, where the whiskers show 95% CI.

The preoperative MHI-5 mean (SD) score was 67 (20) in patients who successfully RTW and 59 (24) in those who failed to RTW (*P* = 0.001; Figure 2). At three months, the MHI-5 score increased by a mean of 13 points (95% CI: 10 to 15) in patients who successfully RTW and remained stable in patients who failed to RTW [increase of two points (95% CI: -2 to 6)]. In all patients, the MHI-5 scores remained stable beyond three months.

### Predictors of RTW

**Figure 3:**
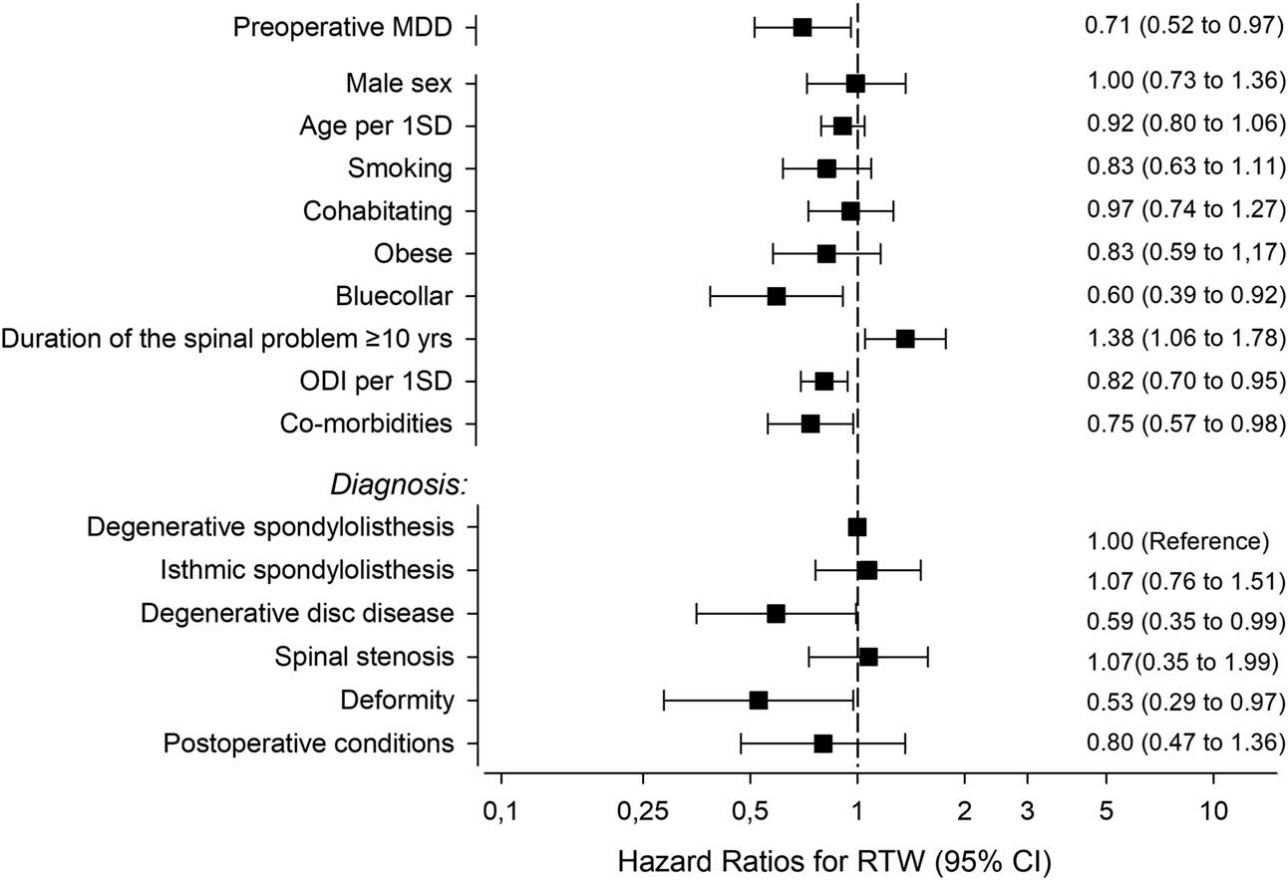
A multivariable analysis evaluating predictors of RTW. *Cohabitating = domestic relationship or marriage; obese = BMI (body mass index) >30; blue-collar = manual worker; duration of spinal problem = 10 years or more; comorbidity = any comorbidity (cardiovascular, diabetes, rheumatoid, psychiatric, and respiratory); postoperative conditions = such as pseudoarthrosis and adjacent-level disease after previous fusion. DDD indicates degenerative disc disease.

In a multivariable analysis (Figure 3), preoperative MDD, lower socioeconomic group, more severe preoperative disability, and the presence of comorbidities decreased the likelihood of RTW. In addition, a very long self-reported duration of spinal complaints (over 10 yr) was predictive of increased RTW.

## DISCUSSION

The present study demonstrated that patients with preoperative MDD were less likely to RTW after LSF surgery. The probability of RTW correlated with the MHI-5 scores until the inflection point of 70, after which a further increase in the MHI-5 score no longer increased the probability of RTW. After LSF, depressive symptoms diminished and did not reappear in those who did successfully RTW. Preoperative MDD, lower socioeconomic group, more severe disability and spinal stenosis as indication for surgery predicted a lower RTW.

The mean MHI-5 score of 65 was lower than that of the general population sample of Finland, with a mean MHI-5 of 71, indicating that depressive symptoms in patients undergoing LSF surgery are overrepresented. Almost half (46%) of the patients demonstrated preoperative MDD according to the threshold of 68 points in MHI-5. In previous studies, the prevalence of preoperative depressive symptoms in patients undergoing LSF was 34% to 35%.^20,21^ Although previous studies used DEPS in the evaluation of depressive symptoms, our study confirms the high prevalence of MDD in LSF patients. Patients undergoing LSF surgery experience long-term pain and disability, factors that may increase the risk of depressive symptoms, and vice versa.^23^

In the present study, preoperative MDD was associated with a lower RTW rate at two years. In a multicenter prospective registry with a two-year follow-up, Zakaria *et al* ^11^ evaluated the presence of depressive symptoms in patients undergoing lumbar fusion using the Patient Health Questionnaire (PHQ-2). In their study, the rate of preoperative MDD was 38% (n = 8585). However, only 456 patients were eligible for RTW analysis at two years, and 56% of the patients with preoperative MDD returned to work, compared with a 73% RTW rate for patients without MDD.^11^ In the present study, the RTW rates of 78% and 87% in the MDD+ and MDD- groups are somewhat higher, respectively, which may mainly be explained by the differences in patient cohort; our patients had undergone fewer previous spine surgeries (27% *vs*. 51%) and had fewer psychiatric comorbidities (3% *vs*. a reported history of depression in 30% and anxiety in 24%). In contrast, the preoperative MDD rate of 46% in our patient cohort is slightly higher than preoperative MDD rate in the study by Zakaria and colleagues, which may be explained by the use of different questionnaires assessing MDD. An interesting finding of the present study was that when the MHI-5 score exceeded 70, it no longer increased the probability of RTW. With scores below 68, the rates of RTW diminished. To the best of our knowledge, this is a novel finding.

In the present study, MHI-5 scores increased above the general population level in patients who resumed working, indicating resolution of depressive symptoms postoperatively. In this group, most of the increase in MHI-5 scores occurred immediately after surgery, with little further improvement beyond three months. Notably, MHI-5 scores did not significantly improve in patients who did not RTW, and the scores did not reach those of patients who successfully resumed work. A study by Zakaria *et al* ^11^ found that 38% of patients who underwent lumbar fusion had MDD preoperatively and only 20% postoperatively. Another study by Wahlman *et al* ^20^ found that the mean DEPS score decreased from 9.4 (SD: 5.9) to 5.9 (SD: 5.6) 1 year after LSF surgery. These results are in line with ours, indicating a relief in depressive symptoms after LSF surgery, although the previous studies did not assess the change in depressive symptoms specifically in relation to RTW.

The multivariate analysis showed that in addition to preoperative MDD, other factors also predicted RTW. Manual workers were less likely to RTW than patients in lighter occupations. Previous studies have shown that a higher physical workload decreased the likelihood of RTW^33–36^. Higher baseline disability was also a negative predictor of RTW. High disability has previously been associated with lower RTW in patients undergoing spine surgery.^33,36,37^ Indication for surgery also predicted RTW, where patients undergoing LSF surgery for spinal stenosis or deformity were less likely to RTW. A previous study with a short three-month follow-up of patients undergoing surgery for lumbar degenerative disease (n = 4694, 26% fusions) did not identify indication for surgery to be a predictor of RTW.^33^ In the present study, patients with any comorbid conditions were also less likely to RTW, but our analysis did not allow for more specific data on these. A retrospective analysis of patients undergoing lumbar spine surgery (n = 22 022, 36% fusions) found no important predictors for RTW in different comorbidities (depression, CAD, anxiety, and diabetes)^34^. An interesting finding in the present study was that patients with a longer duration of spinal complaints (over 10 yr) were more likely to RTW. These results are quite unique, as previous studies have suggested that patients with longer symptom duration either had lower odds to RTW^36^, or that symptom duration was not an important predictor of RTW.^34^ However, these studies had a shorter follow-up period and did not report the effect of a very long duration of the symptoms. A previous systematic review also found that in lumbar spine surgery for chronic back pain, patients with longer chronic pain duration were less likely to RTW, although these patients were not required to be working before surgery, and the follow-up time was at shortest only three months^35^.

### Strengths and Limitations

The primary strength of this study is the prospectively collected, large register database. The study sample represents a population-based cohort of LSF patients, providing real-life data within a national health insurance-based system. Surgical populations and indications for surgery may vary between countries and regions, as there are no universal criteria for LSF surgery. However, the patient selection process accurately reflects the typical patient cohort undergoing LSF surgery in Finland, without specific exclusions. While depressive symptoms were screened only with a self-administered instrument, it has been validated for identifying MDD. Data regarding the patients’ psychiatric background or history of antidepressant use were not at our disposal, which would have allowed for a more comprehensive analysis of depressive symptoms. In addition, it is important to note that our study lacked data on full work ability, making it plausible that not all patients may have returned to work at their full preoperative capacity. During the study period, it was customary to issue a medical certificate allowing sick leave for three months postoperatively. For some occupations, this may seem excessive and can be seen as a limitation to the study.

## CONCLUSIONS

The present study demonstrated that patients with MDD at baseline were less likely to RTW after LSF surgery. After surgery, depressive symptoms diminished in the group who did successfully RTW. Special attention should be paid on patients presenting with depressive symptoms at baseline to facilitate successful RTW after LSF surgery.

Key Points

- Depressive symptoms are overrepresented in patients undergoing LSF surgery.
- Baseline depressive symptoms affect RTW after LSF surgery.
- Depressive symptoms decrease after LSF surgery, especially in patients who successfully RTW.
